# Revisiting Pathogen Exploitation of Clathrin-Independent Endocytosis: Mechanisms and Implications

**DOI:** 10.3390/cells14100731

**Published:** 2025-05-16

**Authors:** Oliver Goldmann, Eva Medina

**Affiliations:** Infection Immunology Research Group, Helmholtz Centre for Infection Research, 38124 Braunschweig, Germany; oliver.goldmann@helmholtz-hzi.de

**Keywords:** clathrin-independent endocytosis, caveolae, bacteria, viruses, dynamin, Cdc42, Arf6, CLIC/GEEC, flotillin

## Abstract

Endocytosis is a specialized transport mechanism in which the cell membrane folds inward to enclose large molecules, fluids, or particles, forming vesicles that are transported within the cell. It plays a crucial role in nutrient uptake, immune responses, and cellular communication. However, many pathogens exploit the endocytic pathway to invade and survive within host cells, allowing them to evade the immune system and establish infection. Endocytosis can be classified as clathrin-mediated (CME) or clathrin-independent (CIE), based on the mechanism of vesicle formation. Unlike CME, which involves the formation of clathrin-coated vesicles that bud from the plasma membrane, CIE does not rely on clathrin-coated vesicles. Instead, other mechanisms facilitate membrane invagination and vesicle formation. CIE encompasses a variety of pathways, including caveolin-mediated, Arf6-dependent, and flotillin-dependent pathways. In this review, we discuss key features of CIE pathways, including cargo selection, vesicle formation, routes taken by internalized cargo, and the regulatory mechanisms governing CIE. Many viruses and bacteria hijack host cell CIE mechanisms to facilitate intracellular trafficking and persistence. We also revisit the exploitation of CIE by bacterial and viral pathogens, highlighting recent discoveries in entry mechanisms, intracellular fate, and host-pathogen interactions. Understanding how pathogens manipulate CIE in host cells can inform the development of novel antimicrobial and immunomodulatory interventions, offering new avenues for disease prevention and treatment.

## 1. Introduction

Endocytosis is a highly complex process by which eukaryotic cells internalize material from the extracellular environment via engulfment in membrane-bound vesicles [1]. This mechanism is essential for nutrient uptake, signaling regulation, and the maintenance of membrane homeostasis. However, many pathogens frequently exploit endocytic pathways to enter host cells [2]. In general, endocytosis begins with the recognition of a specific cargo at the cell surface. The plasma membrane then bends and detaches to form a vesicle that encapsulates the cargo. Finally, systems must be in place to direct these vesicles to their destination and facilitate their fusion with the target membrane. Endocytic processes involving the uptake of large solid particles (>500 nm) are known as phagocytosis [1,3]. This process is primarily carried out by immune cells like macrophages, dendritic cells, and neutrophils. Conversely, smaller particles or fluids are typically engulfed through other endocytic pathways [1,3]. These pathways have traditionally been classified as either clathrin-mediated or clathrin-independent. Clathrin-mediated endocytosis (CME) is characterized by the formation of a clathrin triskelion lattice, which provides structural support for vesicle formation at the plasma membrane [4,5,6]. In addition to clathrin, the CME machinery relies on the coordinated recruitment of over 50 adaptor and scaffolding proteins to form the coated pit [4,5]. Cargo selection and clathrin recruitment during CME are facilitated by adaptor proteins, including the heterotetrameric AP-2 complex, which consists of α, β, µ and σ subunits [7]. Clathrin-independent endocytosis (CIE), on the other hand, does not rely on clathrin structures but instead utilizes alternative mechanisms such as lipid rafts, caveolae, or flotillin [8,9,10,11,12]. CIE generally occurs in lipid rafts at the plasma membrane [13,14]. Lipid rafts are specialized microdomains within the plasma membrane that are enriched in cholesterol, sphingolipids, and proteins [15,16]. These membrane microdomains play a crucial role in organizing and regulating cellular processes, such as signal transduction, membrane trafficking, and protein sorting [15,16]. Lipid rafts are more ordered and tightly packed than the surrounding phospholipid bilayer, making them functionally distinct regions [17].

Several studies have proposed a broad classification of CIE pathways, dividing them into those that rely on a dynamin-mediated scission mechanism (dynamin-dependent) and those that use alternative processes (dynamin-independent) [12,18]. Dynamin is a GTPase enzyme that plays a crucial role in membrane scission during endocytosis. It is primarily involved in the cleavage of vesicles from the plasma membrane, allowing their internalization into the cell. Dynamin assembles around the neck of budding vesicles and, through GTP hydrolysis, constricts and cleaves the membrane, facilitating vesicle release [19]. Dynamin-dependent pathways include caveolae-mediated endocytosis, fast endophilin-mediated endocytosis (FEME), endocytosis regulated by small GTPases such as RhoA and Rac1, and the epidermal growth factor receptor (EGFR) internalization pathway, whereas dynamin-independent pathways include CLIC/GEEC, flotillin-mediated endocytosis (FME), Arf6-mediated endocytosis, and macropinocytosis [19].

Many pathogens, including bacteria and viruses, have developed sophisticated strategies to exploit CIE for entry and survival within host cells. Pathogens often favor CIE over CME for several reasons. For instance, CIE pathways are less likely to be targeted to phago-lysosomes compared to CME, allowing pathogens to evade intracellular killing mechanisms. Additionally, many CIE pathways depend on specific lipid environments or receptors that pathogens can exploit for targeted entry. The diversity of CIE mechanisms provides pathogens with multiple entry routes, increasing their chances of successful infection. Understanding how pathogens exploit CIE is, therefore, critical for the development of targeted therapies. For example, inhibiting specific CIE pathways could prevent pathogen entry without disrupting essential clathrin-dependent processes. Targeting pathogen receptors or lipid raft components could block their ability to hijack CIE. Additionally, modulation of host cell signaling pathways involved in CIE could enhance immune responses against invading pathogens. In summary, CIE serves as a key entry portal for many pathogens. By elucidating the molecular mechanisms underlying pathogen exploitation of CIE, researchers can develop novel strategies to combat infectious diseases. Each of the CIE pathways, along with how bacterial and viral pathogens exploit them to invade host cells, is described in more detail in the following sections.

## 2. Dynamin-Dependent CIE Pathways

### 2.1. Caveolae-Mediated Endocytosis

Caveolae are omega-shaped invaginations of the plasma membrane, measuring 50–100 nm in diameter, and are found in many cell types [20,21,22,23]. They are involved in multiple functions, including cell signaling, lipid regulation, and vesicular trafficking [24,25,26,27]. They also play a key role in cell mechanosensing and mechanoprotection, helping cells to maintain their structural integrity and coordinate appropriate responses to their mechanical environment [28,29]. The structure of caveolae is organized by several components. The caveolar coat consists primarily of transmembrane caveolins (caveolin-1 and caveolin-2), which form a complex that interacts with cytosolic cavins (cavin-1, cavin-2, cavin-3, and cavin-4) [30,31,32,33,34]. These proteins work together to form and stabilize the distinctive bulb-shaped caveolae structures on the plasma membrane. Experiments with knockout mice have demonstrated that caveolin-1 is essential for caveolae formation, whereas caveolin-2 is not required [35]. Cavin proteins are also critical structural components of caveolae [31]. Cavin-1 is essential for proper caveolae formation [36,37,38], while cavin-2, cavin-3, and cavin-4 play regulatory roles that help stabilize caveolae and maintain their functional integrity [39,40,41]. In addition to caveolins and cavins, other supporting proteins localize to the caveolar neck, including Pacsin2 [42,43] and EHD2 [44]. These proteins contribute to stabilizing caveolae at the plasma membrane [33,44,45,46]. Caveolae also contain lipids such as cholesterol, sphingomyelin, and ceramides [47,48]. Cholesterol plays a crucial role in caveolae formation and stability, as their structure is significantly affected by cholesterol depletion or exposure to cholesterol-binding drugs [49,50].

Binding to specific ligands triggers the internalization of caveolae. Although the caveolar endocytic pathway has been shown to play a role in the internalization of several ligands, including albumin [51] and cholera toxin [52], among others, no specific cargo has been found that relies exclusively on caveolae for cellular uptake. The process of caveolar budding is regulated by kinases and phosphatases, including the Src-family tyrosine kinases [53,54,55]. Phosphorylation of caveolin-1 plays a key role in initiating caveolae fission and internalization [54]. Caveolae internalization begins with their detachment from the plasma membrane. Functional studies have shown that caveolae scission is mediated by dynamin-2, with the energy provided by GTP hydrolysis [56,57]. For this reason, caveolae-mediated endocytosis has traditionally been classified as a dynamin-dependent pathway. However, this classification has been challenged by recent studies indicating that dynamin-2 is not required for caveolae formation or fission in HeLa cells but instead functions as an accessory protein that reduces caveolae internalization [58]. Furthermore, cells deficient in all three dynamin isoforms (dynamin-1, -2, and -3 knockout cells) show no significant increase in caveolae abundance and only minor changes in caveolae structure compared to wild-type cells [59]. The discrepancy between studies using functional inhibitors and those employing knockout cells may arise from the limited specificity of dynamin inhibitors. In fact, several inhibitors once thought to specifically target dynamin have been found to affect the actin cytoskeleton instead [60]. Therefore, the role of dynamin in caveolae-mediated endocytosis remains controversial.

Once detached from the plasma membrane, caveolae can be internalized and transported within the cell, most likely through interactions with the cytoskeleton [61,62]. After internalization, caveolae can either fuse with endosomes and subsequently accumulate in lysosomes or follow a non-endosomal pathway to reach intracellular organelles [63]. The interplay between the cargo and caveolae components, whether caveolin itself or one of the associated regulatory kinases and phosphatases, likely plays a significant role in determining the final fate or destination of the cargo. If fusion with early endosomes occurs, it is followed by maturation into late endosomes [64]. Cav-1 has been shown to co-localize with early and late endosomal markers, including Rab5 and Rab7 [64,65].

**Figure 1:**
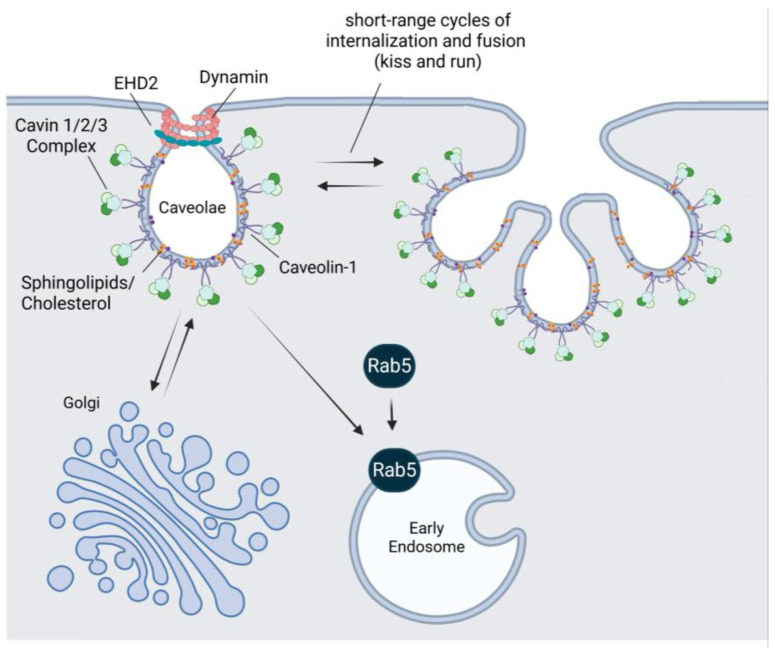
Schematic representation of caveolae-mediated endocytosis. Caveolin-1 and cavins form the structural coat of caveolae. Other proteins, such as dynamin and the dynamin-like ATPase EHD2, along with lipids such as cholesterol and sphingolipids, are also associated with caveolae. During caveolar endocytosis, caveolae bud from the plasma membrane and can be directed to early endosomes or deliver cargo to the Golgi apparatus. Additionally, caveolae can undergo dynamic cycles of internalization and fusion with the plasma membrane without fully committing to deep endocytosis. EHD2, EH domain containing 2. Created with BioRender.com.

Recent technical developments have revealed new pathways for caveolae trafficking beyond the traditional endocytic route. For example, studies demonstrating caveolae-mediated accumulation of cholera toxin in the endoplasmic reticulum (ER) and Golgi apparatus have led to the hypothesis that caveolae may traffic directly from the plasma membrane to the ER [66,67]. In addition, caveolae have been shown to function as specialized platforms that facilitate the transmission of cardioprotective signals to the mitochondria, helping to maintain their optimal function [68,69]. Furthermore, caveolae can undergo dynamic, localized cycles of internalization and fusion with the plasma membrane without fully committing to deep endocytosis, a process sometimes referred to as “kiss-and-run” behavior [70]. This mechanism enables cells to rapidly adjust membrane composition, sense mechanical forces, and regulate signaling without complete vesicle internalization. Figure 1 provides a schematic overview of caveolae-mediated endocytic pathway.

Several bacterial pathogens have been shown to hijack caveolae-mediated pathways to evade host defense mechanisms [71,72,73]. However, most bacteria are larger than 500 nm in diameter, and even some viruses (e.g., vaccinia virus, ~300 nm) significantly exceed the size of caveolae, which typically measure 50–100 nm. Therefore, while caveolae themselves may not directly internalize pathogens, caveolae-associated molecular mechanisms may still contribute to the broader entry process.

### 2.2. Small GTPases-Regulated Endocytosis

Several GTPases, including RhoA, Rac1, Cdc42, and RhoG, have also been shown to regulate CIE [74,75,76,77]. The internalization of the β-chain of the interleukin-2 receptor (IL-2R-β) involves dynamin and relies on the small GTPase RhoA [78,79]. Upon ligand binding, IL-2R-β localizes to detergent-resistant membranes, a characteristic typically associated with CIE mechanisms [78,79].

Cdc42 regulates the endocytosis of GPI-anchored proteins (GPI-APs), a process independent of clathrin and caveolin [74]. The endocytosis of GPI-APs and IL-2R-β is distinctly regulated by Rho family proteins. While IL-2R-β endocytosis depends on RhoA and Rac1 but not Cdc42, the endocytosis of GPI-APs is governed by different regulatory mechanisms [78].

### 2.3. Fast Endophilin-Mediated Endocytosis (FEME)

Fast endophilin-mediated endocytosis (FEME) is a dynamin-dependent CIE pathway regulated by endophilin [80,81]. Endophilin proteins contain both an SH3 (Src homology 3) domain and a BAR (Bin-Amphiphysin-Rvs) domain [82,83]. Endophilin induces plasma membrane curvature via the BAR domain, positions cargo via the SH3 domain, and facilitates membrane scission by recruiting dynamin and actin [80,81,84,85]. FEME is a rapid process capable of transporting a wide range of cargo, including receptors such as β1- and α2A-adrenergic receptors, dopamine receptors, tetrameric IL-2R, PlexinA1, and cholera and Shiga toxins [80,81].

**Figure 2:**
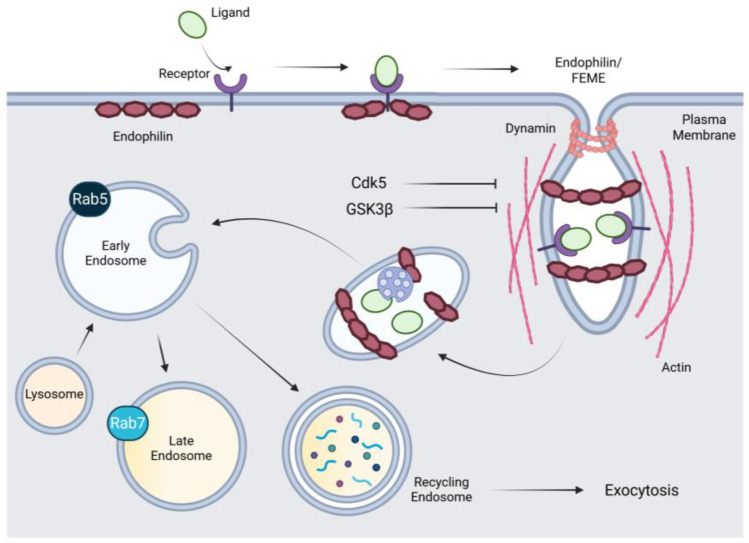
Schematic representation of fast endophilin-mediated endocytosis (FEME). Upon ligand binding to specific receptors, endophilin is recruited to the plasma membrane, where it induces membrane curvature to form tubular invaginations. This process triggers immediate vesicle scission through the help of dynamin and actin polymerization. The resulting vesicles are then transported into the cell, where the cargo is sorted for recycling back to the plasma membrane, degradation in lysosomes, or signaling from the endosome. Cdk5 and GSK3β are kinases that negatively regulate FEME. Dsk5, cyclin-dependent kinase 5; GSK3β, glycogen synthase kinase-3 beta. Created with BioRender.com.

FEME is inactive by default and is activated only when specific cell surface receptors are stimulated by their corresponding ligands [81]. The rapid activation of FEME upon receptor stimulation is triggered by a cascade of molecular events initiated by Cdc42 [80]. GTP-loaded Cdc42 recruits Cdc42-interacting protein 4 (CIP4) and formin-binding protein 17 (FBP17), which interact with SH2-containing inositol phosphatase 2 (SHP2) and lamellipodin [86]. Endophilin binds to the proline-rich region of lamellipodin, leading to the accumulation of endophilin in clusters at specific sites on the plasma membrane [86]. Upon activation, the receptors are rapidly targeted to pre-existing endophilin clusters, which then bud to form FEME carriers in the cytosol. Membrane scission of the carriers requires the coordinated action of dynamin, actin, and the BAR domain of endophilin [84,85]. FEME carriers move quickly to fuse with early endosomes and efficiently deliver their cargo. The entire process takes place within 5–10 s [86]. FEME is negatively regulated by Cdk5 and GSK3β [87]. These kinases antagonize the binding of endophilin to dynamin, thereby inhibiting membrane scission and the transport of FEME carrier onto microtubules [87]. Cdk5 and GSK3β may also exert additional regulatory effects, either by controlling other critical steps in the FEME pathway or, more indirectly, by influencing the activity of other kinases [87]. A schematic representation of the FEME pathway is shown in Figure 2.

### 2.4. Clathrin-Independent Internalization of the Epidermal Growth Factor Receptor (EGFR)

EGFR is a receptor tyrosine kinase that is activated by ligands such as epidermal growth factor (EGF) [88,89]. Upon activation, EGFR undergoes dimerization, autophosphorylation, followed by internalization into the cell, where it regulates downstream signaling and cellular processes such as proliferation, cell differentiation, and development [88,89].

While CME is the dominant mechanism for EGFR internalization at low EGF concentrations (1 ng/mL), allowing receptor recycling back to the cell membrane, CIE plays a significant role for EGFR internalization at high physiological EGF concentrations (20–100 ng/mL), targeting EGFR to the lysosome for degradation [90,91]. Thus, CIE may act as a suppressor of EGFR signaling under conditions of excessive stimulation.

The EGFR CIE endocytic pathway is cholesterol- and dynamin-dependent and requires EGFR ubiquitination, along with the involvement of proteins containing ubiquitin-binding domains [90]. Once ubiquitinated, EGFR is targeted to lysosomes for degradation [92]. The ER-resident protein reticulon-3 (RTN3) is a key regulator of EGFR CIE endocytic pathway [89]. RTN3 facilitates contact sites between the ER and the plasma membrane, which are essential for initiating membrane tubulation and invagination. Upon EGF binding, EGFR activates the inositol triphosphate receptor (IP3R), triggering calcium release from the ER. This calcium release stabilizes the membrane tubules thereby promoting EGRF internalization [91]. Additionally, CD147 represents at least one further cargo that is transported along the same CIE pathway as EGFR [91].

## 3. Dynamin-Independent CIE Pathways

It is now widely recognized that several CIE pathways exist that do not rely on dynamin for membrane scission [93,94]. A common feature of dynamin-independent CIE pathways is the involvement of small GTPases, particularly the Rho family member Cdc42 or the Arf family member Arf6 [93]. These pathways are described in more detail in the following sections.

### 3.1. CLIC/GEEC Pathway

The CLIC/GEEC pathway is a CIE mechanism characterized by the formation of uncoated tubulovesicular structures known as clathrin-independent carriers (CLIC) [74]. CLIC formation involves the coordinated assembly of Arf1, the BAR-domain protein IRSp53 (Insulin receptor substrate p53), which binds to actin, and the Arp2/3 complex at the plasma membrane. Upon activation by Cdc42, IRSp53 triggers Arp2/3-mediated actin polymerization [95], a process that facilitates CLIC detachment from the cell membrane. CLIC deliver their cargo to early endocytic compartments called GPI-AP-enriched compartments (GEEC), which are rich in glycosylphosphatidylinositol-anchored proteins (GPI-APs) [74,96]. The GEEC subsequently fuse with sorting endosomes in a process dependent on the small GTPase Rab5 and phosphoinositide 3-kinase (PI3K) activity [96]. Since CLIC and GEEC pathways occur sequentially, the process is referred to as the CLIC/GEEC pathway [97]. This endocytic route mediates the uptake of specific types of cargo, including GPI-Aps, fluid phase markers, and certain receptors such as integrins. Glycosylated cargo proteins such as CD44 and α5β1 integrin, which are involved in cell adhesion and migration, can bind to lectins like galectin-3, which may be either membrane-bound on the cell surface or freely diffusible in the extracellular space [98]. This binding promotes the clustering of glycosylated proteins and glycolipids in the cell membrane, leading to membrane curvature and vesicle formation [99]. Cargo internalized via GEEC may then be sorted either for recycling back to the plasma membrane or directed to late endosomes/lysosomes for degradation.

**Figure 3:**
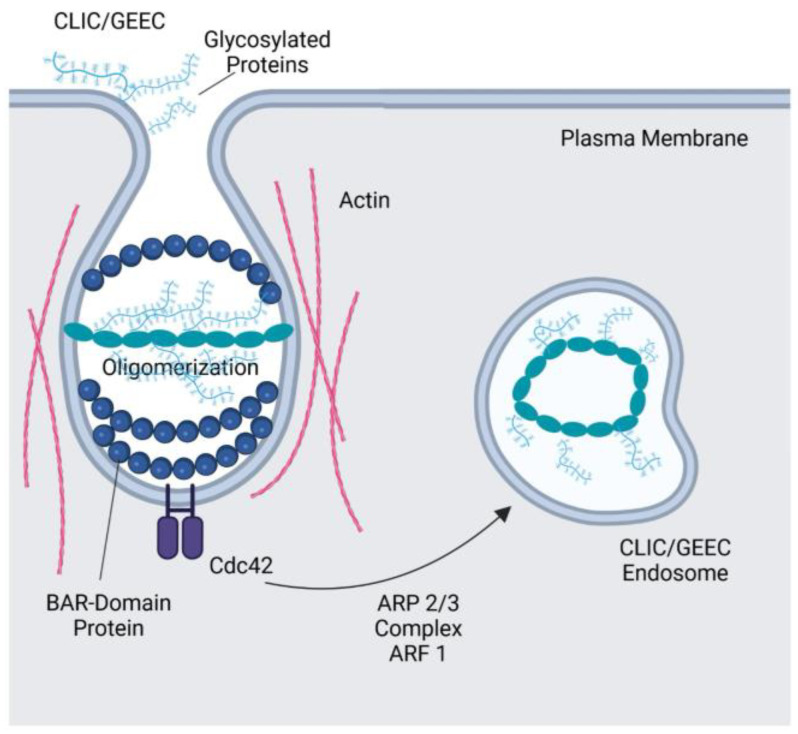
Schematic representation of CLIC/GEEC pathway. The CLIC/GEEC pathway is mediated by uncoated tubulovesicular primary carriers called clathrin-independent carriers (CLIC) that arise directly from the plasma membrane and subsequently mature into tubular early endocytic compartments known as glycosylphosphotidylinositol-anchored protein (GPI-AP)-enriched compartments (GEEC). This pathway is regulated by the Rho family GTPase Cdc42 and actin dynamics via the ARP2/3 complex. Cdc42 activates the ARP2/3 complex, which facilitates the formation of branched actin networks necessary for membrane curvature and vesicle formation. BAR-Domain, Bin-Amphiphysin-Rvs domain; ARF1, ADP-ribosylation factor 1; ARP2/3, actin related protein 2/3. Created with BioRender.com.

Cdc42, a member of the Rho family of GTPases, is a key regulator of the CLIC/GEEC pathway and influences the formation, trafficking, and maturation of endocytic vesicles through its interactions with the actin cytoskeleton and endocytic machinery [74,75]. GPI-APs at the plasma membrane are arranged into cholesterol-dependent nanoscale clusters, a process driven by cortical actin activity [100]. Indeed, recruitment of the actin polymerization machinery by cholesterol-sensitive Cdc42 activation is essential for the GEEC pathway [75]. The cycling of Cdc42 between its active (GTP-bound) and inactive (GDP-bound) states at the plasma membrane is essential for the recruitment of the actin polymerization machinery in the CLIC/GEEC pathway. This process is regulated by GBF1, a guanine nucleotide exchange factor (GEF) that activates Arf1 [76]. The activated Arf1 protein recruits the Rho GTPase-activating protein 10 (ARHGAP10), which inactivates Cdc42 and returns it to its cycling state. Another key regulator of Cdc42 and the CLIC/GEEC pathway is GTPase regulator associated with focal adhesion kinase 1 (GRAF1). GRAF1 contains a RhoGAP domain that inactivates Cdc42, as well as a BAR domain and an SH3 domain that contribute to its function in membrane remodeling and endocytosis [101]. Figure 3 illustrates a schematic representation of the CLIC/GEEC pathway.

### 3.2. Arf6-Dependent Endocytosis

Another CIE pathway that does not rely on dynamin for vesicle scission is associated with the small GTPase ADP-ribosylation factor 6 (Arf6) [102,103,104]. Arf6 regulates a CIE pathway, in which cargo is initially internalized into Arf6-enriched vesicles and later has the potential to be recycled back to the plasma membrane [102,105]. Arf6 plays a key role in the endocytosis of several integral membrane proteins that lack adaptor protein recognition sequences [104]. It is also involved in the internalization and recycling of plasma membrane proteins involved in cell adhesion, such as cadherins and integrins, as well as proteins involved in the immune response, including major histocompatibility complex class I (MHC-I), and certain GPI-anchored proteins such as CD55 and CD59 [106]. Arf6-mediated endocytosis is increasingly recognized as a key trafficking pathway that plays a crucial role in regulating cell adhesion, migration, tumor invasion, and cytokinesis through the modulation of actin cytoskeleton reorganization [102,103,107].

Like other GTPases, Arf6 switches between an active state when bound to GTP and an inactive state when bound to GDP. Its activation is regulated by two distinct classes of proteins: guanine nucleotide exchange factors (GEFs), which facilitate the exchange of GDP for GTP to activate Arf6, and GTPase-activating proteins (GAPs), which promote the hydrolysis of GTP, returning Arf6 to its inactive GDP-bound form [104,108]. Inactivation of Arf6 shortly after internalization is necessary to ensure proper sorting of the endosomal cargo. Overexpression of the constitutively active form of Arf6 disrupts this process, causing cargo to become trapped in internal vacuolar structures. These structures are coated with phosphatidylinositol 4,5-bisphosphate (PIP_2_), highlighting the critical role of Arf6 inactivation in normal endosomal trafficking [109]. As mentioned above, in the Arf6-regulated CIE pathway, cargo is initially internalized and transported within Arf6-enriched vesicles before being recycled back to the plasma membrane. The hydrolysis of Arf6-GTP to Arf6-GDP, along with the depletion of PIP_2_, is essential for these vesicles to fuse with sorting endosomes [110,111]. During the initial phase of this endocytic pathway, Rab35 associates with newly endocytosed vesicles and recruits OCRL (Oculocerebrorenal Syndrome of Lowe) protein, an enzyme that degrades PIP_2_ [112]. Rab35 also plays a role in the regulation CIE vesicles, potentially facilitating the inactivation of Arf6 and the hydrolysis of PIP_2_ [113]. Consequently, Arf6 and Rab35 function sequentially to ensure the proper internalization and early sorting of cargo.

### 3.3. Flotillin-Mediated Endocytosis (FME)

Flotillin-mediated endocytosis (FME) is a CIE pathway involving flotillin proteins associated with endocytic vesicles [114,115,116,117]. The flotillin family includes flotillin-1 (or reggie-2) and flotillin-2 (or reggie-1), which belongs to the SPFH (stomatin/prohibitin/flotillin/HflK/C) domain-containing proteins group [118]. Like other SPFH domain-containing proteins, flotillins tend to form both hetero- and homo-oligomers [119,120]. The assembly of flotillins into microdomains induces membrane curvature, promotes the formation of plasma membrane invaginations, and facilitates the development of intracellular vesicles [121]. Flotillins are ubiquitously expressed in mammalian cells [122] and play a role in the endocytosis of molecules such as glycosylphosphatidylinositol (GPI)-linked proteins, the cholera toxin B subunit, and glycosphingolipids [123,124]. Flotillins do not span the cytoplasmic membrane but are instead anchored to the cytosolic leaflet of the plasma membrane via fatty acid modifications [125]. FME is regulated by the Src family tyrosine kinase Fyn and probably by other Src kinases [116,126].

### 3.4. Macropinocytosis

Macropinocytosis is a cellular process in which cells engulf large amounts of extracellular material, including nutrients, antigens, and pathogens, through the formation of large vesicles called macropinosomes [127,128,129]. This pathway plays a crucial role in various physiological processes, including nutrient uptake, signaling, antigen presentation, and cell migration [127,128]. The cups and ruffles involved in macropinocytosis are formed and extended through actin polymerization, a dynamic process driven by the cytoskeleton [129]. Actin filaments assemble and push the plasma membrane outward, creating protrusions such as ruffles or cups [129]. As these actin-driven ruffles fold back onto the plasma membrane, they engulf extracellular material, forming macropinosomes [129]. This process heavily depends on the reorganization of the actin cytoskeleton, which is regulated by signaling pathways involving proteins such as Arp2/3, SCAR/WAVE, Rac1, PI3K, and Ras [128,130]. PI3-kinase are generally essential for micropinocytosis, primarily through their role in generating PIP3 and coordinating actin-driven membrane remodeling [131,132]. PI3K activity is essential for priming ruffle membranes to seal into macropinosomes [133].

Once formed, the macropinosome matures and interacts with other cellular compartments to process its contents [130]. Actin polymerization is, therefore, a crucial mechanism that enables the initiation and progression of macropinocytosis. In addition to the Rac1, PI3K, and Ras proteins, several Rab proteins, including Rab5, Rab20, Rab21, and Rab34, as well as Arf proteins such as Arf6 and Arf1, are involved in this process [130].

Macropinocytosis can also be exploited by pathogens such as bacteria, viruses, protozoa, and prions to invade host cells and evade the host immune system [134,135]. Examples include *Salmonella* [136], *Shigella* [137], *Chlamydia* [138] *Brucella* [139], *Mycobacterium* spp. [140,141], *Legionella* [142], *E. coli* [143], Vaccinia virus [144], and HIV-1 [145].

### 3.5. Convergence and Crosstalk Between CIE Pathways

Although the CIE pathways are quite diverse, many of them share regulatory components and cargo specificity, leading to functional overlap. Several CIE pathways, including caveolae-mediated uptake, Arf6-associated endocytosis, and the CLIC/GEEC pathway, as well as certain non-caveolar, lipid raft-dependent carriers (some of which may be linked to CLIC), rely on membrane cholesterol. These pathways are disrupted by cholesterol depletion, highlighting their reliance on cholesterol-binding proteins such as flotillins [121] and caveolins [146]. Cholesterol also plays a key role in regulating Cdc42, a small GTPase critical for the CLIC/GEEC pathway [147]. The distinction between the CLIC/GEEC pathway and macropinocytosis is not always clear-cut, as they appear to share some molecular machinery [3]. Furthermore, there is significant cross-talk between different endocytic pathways—for instance, altering the expression of caveolar proteins can influence CLIC/GEEC-mediated uptake [148]. Interestingly, blocking one endocytic pathway can trigger compensatory changes in others. For example, when dynamin is inhibited (such as through a temperature-sensitive mutant), cells rapidly upregulate dynamin-independent uptake mechanisms [149]. Moreover, the same cargo can be internalized via multiple endocytic pathways. For instance, cholera toxin can enter cells via clathrin-coated pits, caveolae, and a major clathrin- and caveolin-independent pathway [150]. The dominant pathway often depends on cell type.

## 4. Exploitation of CIE Pathways by Pathogens for Host Cell Entry and Infection

Several pathogens hijack CIE pathways to enter host cells, evading immune detection and ensuring their survival. The next sections showcase prominent bacterial and viral pathogens that exploit CIE for host cell invasion.

### 4.1. Exploitation of CIE by Bacterial Pathogens

Several bacterial pathogens co-opt CIE pathways to invade, persist and alter host cell functions to their advantage [2,72,151,152]. Not only do intracellular bacterial pathogens use CIE to invade their host cells to avoid degradative pathways, but there is also increasing evidence that extracellular bacteria can exploit CIE to internalize into host cells [2,72,151,152]. Entry into cells via CIE is thought to protect extracellular bacteria from the immune response and the bactericidal effects of antibiotics. The following sections discuss some of the most notable examples of extracellular and intracellular bacteria that use CIE to invade host cells (Table 1).

#### 4.1.1. *Listeria monocytogenes*

**Figure 4:**
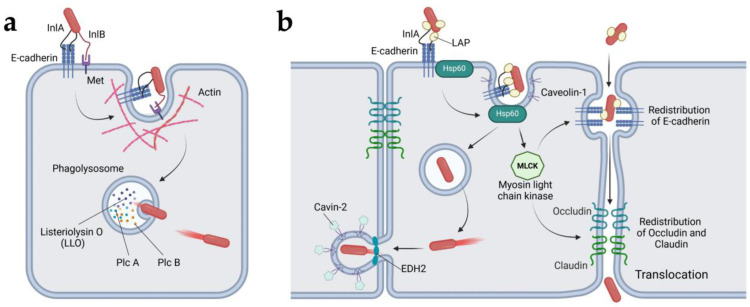
Mechanisms of adhesion, invasion, and translocation of epithelial cells by *L. monocytogenes*. (**a**) *L. monocytogenes* binds to epithelial cell receptors E-cadherin and the Met via InlA and InlB. This interaction triggers receptor-mediated endocytosis of *L. monocytogenes*. Once inside the cell, *L. monocytogenes* uses LLO, PlcA, and PlcB to escape from the endocytic vesicle into the cytoplasm. (**b**) The Listeria adhesion protein (LAP) interacts with the epithelial cell surface receptor Hsp60, facilitating access of InlA to basolateral E-cadherin. This interaction triggers caveolin-1-mediated endocytosis, activates MLCK, and disrupts the intestinal cell-cell barrier by redistributing junctional proteins such as claudin-1, occludin, and E-cadherin, ultimately enabling bacterial translocation across the epithelium. InlA, internalin A; InlB, internalin B; E-cadherin, epithelial cadherin; LLO, listeriolysin O; LAP, Listeria adhesion protein; Hsp60, heat shock protein 60; Met, MET receptor tyrosine kinase; PlcA, phosphatidylinositol-specific phospholipase; PlcB, broad-range phospholipase C, MLCK, myosin light chain kinase. Created with BioRender.com.

*Listeria monocytogenes* is a pathogenic foodborne bacterium that causes listeriosis, a serious infection that mainly affects pregnant women, newborn babies, the elderly, and people with weakened immune systems [180]. The primary route of infection for *L. monocytogenes* is through the intestinal tract. Overcoming this barrier is a critical first step for the bacterium to invade and spread to deeper tissues in the body [181,182]. *L. monocytogenes* is a facultative intracellular pathogen with the ability to actively invade and replicate in mammalian cells, including macrophages and epithelial cells [183,184]. To enter host epithelial cells, bacterial surface proteins such as internalin A (InlA) and internalin B (InlB) bind to specific cellular receptors such as E-cadherin and the Met receptor tyrosine kinase (also called hepatocyte growth factor receptor, HGFR) [185,186,187] (Figure 4a). This interaction triggers signaling pathways that result in the pathogen being engulfed and enclosed in a tight membrane-bound vesicle [183,184]. Once inside the cell, *L. monocytogenes* uses the pore-forming toxin listeriolysin O and the phospholipases PlcA and PlcB, which are encoded by the *plcA* and *plcB* genes located in Listeria pathogenicity island 1 (LIPI-1), to escape from the endocytic vesicle and enter the cytoplasm [188,189]. There, *L. monocytogenes* replicates and uses F-actin-based motility to spread from one cell to another [190]. Although *L. monocytogenes* internalization via internalins has been shown to require the recruitment of clathrin [191,192], caveolin-1-mediated endocytosis and a specific group of caveolar proteins, including caveolin-1, cavin-2, and EHD2, have been shown to be critical for efficient bacterial cell-to-cell spreading [153]. Thus, that *L. monocytogenes* appears to exploit a caveolin-1-mediated endocytic pathway to facilitate its movement between epithelial cells [153].

Another route of *L. monocytogenes* internalization, independent of internalins, has been identified and appears to play a crucial role in the bacterium translocation across the intestinal barrier. This pathway is mediated by the Listeria adhesion protein (LAP) [154] (Figure 4b). In this process, *L. monocytogenes* uses caveolin-1-mediated endocytosis to internalize integral apical junctional proteins and target them to early and recycling endosomes, facilitating bacterial translocation across epithelial cells. Additionally, the interaction of LAP with its cognate receptor, Hsp60, has been shown to induce the endocytosis of junctional proteins that are essential for InlA to access basolateral E-cadherin [154]. LAP interacts directly with Hsp60 to trigger canonical NF-κB signaling, which promotes the activation of myosin light chain kinase (MLCK). This process leads to the opening of the intestinal cell-cell barrier by redistributing key junctional proteins, including claudin-1, occludin, and E-cadherin, within the cells, ultimately facilitating bacterial translocation [193]. Therefore, the cooperation between LAP and InlA facilitates the translocation of *L. monocytogenes* across the intestinal epithelial barrier.

#### 4.1.2. *Mycobacterium tuberculosis*

*Mycobacterium tuberculosis* is the etiological agent of tuberculosis, an infection that affects a quarter of the human population and is associated with high mortality rates [194]. *M*. *tuberculosis* is an obligate human pathogen with no known environmental reservoir [195]. Following phagocytosis by macrophages, the pathogen resists intracellular killing mechanisms, allowing it to survive and replicate within these cells [196,197]. To ensure its survival, *M. tuberculosis* has developed strategies to evade, manipulate, and exploit the host immune defenses, turning these mechanisms to its advantage [198,199]. Normally, when bacteria are phagocytosed by macrophages, they are rapidly eliminated in phago-lysosomes. However, mycobacteria persist in specialized compartments known as mycobacterial phagosomes, which do not acquire lysosomal hydrolases or an acidic environment, which are essential for pathogen degradation [200].

**Figure 5:**
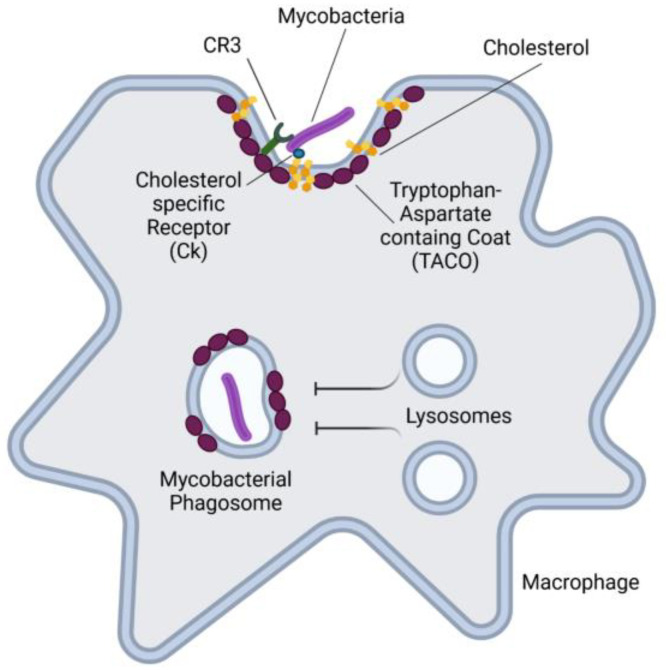
Schematic representation of *M. tuberculosis* invasion of macrophages. *M. tuberculosis* binds to specific receptors, such as CR3, to enter the macrophage. TACO is recruited via cholesterol to the phagosome, stabilizing it and preventing fusion with lysosomes. This interaction protects mycobacteria from degradation. *M. tuberculosis* also expresses a cholesterol-specific receptor, Ck, which facilitates its entry into macrophages. CR3, complement receptor 3; TACO, tryptophan-aspartate-containing coat: Ck, cholesterol specific receptor. Created with BioRender.com.

*M. tuberculosis* can bind to specific receptor molecules, including complement receptor type 3 (CR3), to enter the host cells [197,201,202] (Figure 5). CR3 facilitates the entry of mycobacteria into macrophages without triggering their activation [155]. Cholesterol plays a crucial role in recruiting the tryptophan-aspartate-containing coat (TACO) to the phagosome [155,203]. This interaction helps protect mycobacteria from degradation, allowing them to survive in host cells.

In addition to interacting with cell surface receptors, *M. tuberculosis* can also directly interact with cholesterol in plasma membrane lipid rafts [155] (Figure 5). Depletion of cholesterol has been shown to reduce the ability of *M. tuberculosis* to enter host cells [155]. Furthermore, the requirement for cholesterol to facilitate stable binding suggests that *M. tuberculosis* possesses a high-affinity cholesterol binding site on its surface. In this context, it has been reported that *M. tuberculosis* expresses a cholesterol-specific receptor, known as Ck, which mediates mycobacterial entry into macrophages [156]. Additionally, Ck has been shown to regulate the expression of the gene encoding TACO, thereby influencing the survival of *M. tuberculosis* within macrophages [156].

Using caveolin-1-deficient mice, Wu et al. [158] reported that caveolin-1 plays a role in the early clearance of *Mycobacterium bovis* Bacillus Calmette-Guérin (BCG), an attenuated mycobacteria often used as a model to study the pathogenicity of *M. tuberculosis*. Although caveolin-1 does not affect phagocytosis of BCG, it influences intracellular bacterial killing, most likely by regulating acid sphingomyelinase-dependent ceramide production [158]. Supporting this, sphingomyelinase/ceramide has been implicated in the internalization and killing of a variety of pathogens [204].

Although macrophages are the primary host cells for *M. tuberculosis*, other cell types, including mast cells [205], have also been shown to internalize *M. tuberculosis*. Mast cells are traditionally known for their role in allergic reactions and defense against parasites, but emerging evidence suggests they may also play a role in the immune response to bacterial infections, including mycobacteria [206]. Internalization of *M. tuberculosis* into mast cells is mediated by a lipid rafts and is cholesterol-dependent [157]. Once internalized, *M. tuberculosis* can survive within mast cells, similar to its ability to persist in macrophages [157]. This may provide a niche for mycobacteria to evade immune detection [157].

#### 4.1.3. *Streptococcus pyogenes*

*Streptococcus pyogenes*, also known as Group A Streptococcus, is an important human pathogen responsible for infections that can range from mild, such as pharyngitis and impetigo, to very severe, such as necrotizing fasciitis [207]. Although classified as an extracellular pathogen, numerous studies have demonstrated the ability of *S. pyogenes* to internalize and survive within host cells [208,209,210,211]. By surviving intracellularly, *S. pyogenes* evades immune defense mechanisms such as antibodies or complement-mediated killing, as well as the effects of antibiotics with limited ability to penetrate host cells [212,213]. This allows the bacteria to persist in a latent state and potentially contribute to recurrent infections [214,215].

*S. pyogenes* uses various pathways to internalize into host epithelial cells [159,213]. Based on the observation that *S. pyogenes* co-localizes with caveolin-1 and that disruption of lipid rafts or cholesterol depletion inhibits its invasion into human epithelial HEp-2 cells, Rohde et al. [159] proposed that *S. pyogenes* utilizes the caveolae pathway to enter host cells. They demonstrated that the streptococcal fibronectin-binding protein I (SfbI), expressed on the surface of *S. pyogenes*, plays a key role in bacterial invasion through the caveolae-dependent pathway [159]. The mechanism involves SfbI interacting with fibronectin, which serves as a bridge to the α5β1 integrin on the host cell membrane [216]. This interaction triggers integrin clustering and activates signaling cascades, resulting in the formation of caveolae that facilitate bacterial internalization [159]. By exploiting the caveolae-mediated internalization pathway, *S. pyogenes* bypasses the conventional endosomal-lysosomal route that typically leads to pathogen destruction, thereby ensuring its survival. In contrast to these observations, other studies using genetic knockdown or silencing of caveolin-1 in host cells have shown that caveolae are not involved in the internalization of *S. pyogenes* into epithelial cells, irrespective of SfbI expression [217]. The authors of that study also reported that caveolin-1 is not required for bacterial internalization but instead inhibits *S. pyogenes* internalization into host cells through a process independent of caveolae formation [217]. The exact mechanism was not identified. The reason for the discrepancy between these studies remains unclear and has not yet been determined.

#### 4.1.4. *Staphylococcus aureus*

*Staphylococcus aureus* is a major human pathogen responsible for significant global morbidity and mortality, a situation worsened by the propensity of the bacterium to develop drug resistance [218,219]. *S. aureus* can cause a wide range of infections, from minor skin infections to serious invasive diseases, including pneumonia, osteomyelitis, bacteremia, and endocarditis [218]. *S. aureus* has evolved multiple mechanisms to evade the host immune response, including the ability to internalize and survive within host cells [220,221,222]. The capacity of *S. aureus* to survive within host cells is a key factor contributing to its antibiotic tolerance and treatment failure [223]. *S. aureus* can invade and survive inside various types of host cells, including phagocytic cells, epithelial cells, keratinocytes, and endothelial cells [223]. The interplay between *S. aureus* and host cells is remarkably complex and may differ depending on the type of host cell involved.

**Figure 6:**
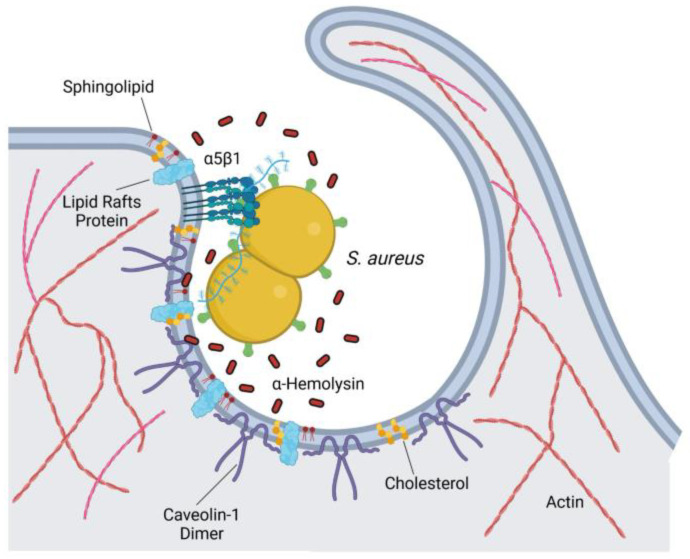
Schematic representation of the mechanism used by *S. aureus* to internalize into human respiratory epithelial cells. *S. aureus* attaches to α5β1 integrins on the surface of respiratory epithelial cells releases α-hemolysin, which directly interacts with caveolin-1. This interaction destabilizes the cell membrane, facilitating *S. aureus* endocytosis. Created with BioRender.com.

Our group recently demonstrated that *S. aureus* utilizes caveolin-1 and lipid raft-mediated endocytosis to internalize into human respiratory epithelial cells, which are likely to be the first host cells to encounter *S. aureus* in the respiratory tract [160]. Pharmacological disruption of lipid rafts or inhibition of caveolin-1 function in lung epithelial cells significantly reduces *S. aureus* internalization [160]. The cytotoxin a-hemolysin, one of the major virulence factors involved in the pathogenesis of *S. aureus* infections [224], appears to be critical for bacterial internalization, as evidenced by the failure to of a mutant *S. aureus* strain deficient in a-hemolysin expression to internalize [160]. As the ability of a-hemolysin to interact with caveolin-1 has been extensively documented [225,226,227,228,229], the interaction between a-hemolysin, released by *S. aureus*, and caveolin-1 on the lipid rafts may trigger *S. aureus* endocytosis [160]. In this regard, Hoffmann et al. [230] found that in fibroblasts, caveolin-1 acts as a stabilizing factor, maintaining the structure of the plasma membrane within lipid rafts. *S. aureus* attachment to a5b1 integrins on the surface of the respiratory epithelial cells is a prerequisite for bacterial internalization [160]. Thus, *S. aureus* attached to the surface of epithelial cells releases a-hemolysin, which directly interacts with caveolin-1, resulting in destabilization of the cell membrane and the initiation of *S. aureus* endocytosis (Figure 6).

#### 4.1.5. *Escherichia coli*

Although *Escherichia coli* is normally a harmless commensal bacterium, certain isolates have been implicated in a wide range of serious infections. These include enteropathogenic *E. coli* (EPEC), enterohaemorrhagic *E. coli* (EHEC) and uropathogenic *E. coli* (UPEC) [231]. UPEC is a leading cause of urinary tract infections (UTIs) [232], which are among the most common bacterial infections worldwide [233]. A characteristic of UTIs is their tendency to recur [234]. The ability of *E. coli* to invade and replicate within host cells has been proposed as an important factor in recurrent and chronic UTIs [235]. UPEC expresses a variety of virulence factors, including type 1 fimbriae, P fimbriae, S fimbriae, F1C fimbriae, Dr fimbriae, curli fibers, and PapC, which are required for colonizing the bladder and invading host cells [231,232]. Fimbriae allow UPEC to attach to bladder cells in the urinary tract and resist mechanical expulsion from the urinary system during urination.

The fimbrial adhesin FimH, a type I fimbriae located at the tip of phase-variable type 1 pili, is one of the best characterized adhesins of UPEC [236,237]. UPEC can not only adhere to bladder cells but also invade them [236,237,238]. The intracellular environment may help UPEC evade the forceful flow of urine in the bladder and potentially protect them from the effects of antibiotics and immune defenses. UPEC can also replicate intracellularly, forming a bacterial reservoir within the bladder that may act as a source of recurrent acute infections [239]. Several mechanisms have been identified by which UPEC enter host cells. These include the exploitation of host Rho GTPases via the secreted toxin CNF1 [240], and manipulation of host complement receptors [241]. Additionally, Martinez et al. [236] demonstrated that FimH also acts as an invasin, facilitating UPEC invasion by inducing rearrangement of the host cell cytoskeleton. FimH has been shown to mediate the invasion and translocation of extraintestinal pathogenic *E. coli* across the intestinal epithelium [242]. Several host cell receptors have been identified that interact with FimH, including glycosylated uroplakin Ia (UP1a) [243], members of the carcinoembryonic antigen-related cell adhesion molecule (CEACAM) family [244], the glycosylphosphatidylinositol (GPI)-anchored protein CD48 [166], and β1 and α3 integrins [162]. Rho GTPases, lipid rafts, and caveolin-1 also contribute to the invasion process [161,163]. Once internalized into terminally differentiated superficial bladder epithelial cells, UPEC rapidly replicates and assemble into biofilm-like structures known as intracellular bacterial communities (IBCs) or pods, which provide temporary protective niches [245].

Afa/Dr diffusely adhering *E. coli* (DAEC) is a pathotype associated with UTIs and diarrhea in children [164]. This pathotype expresses Afa/Dr adhesins, which mediate invasion of polarized epithelial cells through interaction with the α5β1 integrin and a pathway involving caveolae and microtubules [165].

*E. coli* can also internalize into bone marrow-derived mast cells [167]. As mentioned above, mast cells are tissue-resident immune cells located at the interfaces between the body and the environment, contributing to the first line of immune defense against invading pathogens [206]. The internalization of *E. coli* into mast cells appears to be mediated by CD48, a receptor for FimH, which is present on caveolae [167]. Since caveolae do not fuse with endosomes, *E. coli* exploits the caveolar compartments to evade the bactericidal activity of mast cells and remain viable within these long-live cells [167].

*E. coli* can also cause meningitis, particularly in neonates [246]. While most cases result from hematogenous spread, the exact mechanism by which circulating *E. coli* crosses the blood-brain barrier remains unclear [247]. In this context, Sukumaran et al. [168] reported that *E. coli* internalization into human brain microvascular endothelial cells occurs via caveolae. The interaction of caveolin-1 with phosphorylated protein kinase C alpha (PKCa) at the *E. coli* attachment site, along with the integrity of cholesterol-enriched microdomains, appears to be critical for the invasion process [168].

#### 4.1.6. *Salmonella typhimurium*

*Salmonella enterica* serovar Typhimurium (*S. typhimurium*) is a leading cause of food- and waterborne infections worldwide [248]. Infection begins when contaminated food or water is consumed, allowing *Salmonella* to reach the intestinal lumen and cause gastrointestinal disease [248]. In some individuals, the infection progresses as the bacteria penetrate the intestinal lining and then spread throughout the body [248]. *Salmonella* is a facultative intracellular pathogen capable of invading non-phagocytic host cells [249]. The ability to invade these cells is considered to be a crucial step in the development of *Salmonella* infections.

One of the best studied routes for *Salmonella* spread from the intestinal lumen is through the microfold (M) cells on Peyer’s patches [250]. *Salmonella* uses a type III secretion system (T3SS), encoded by the pathogenicity island 1 (SPI-1), to inject effector proteins directly into the host cell [251]. These effector proteins, such as SipA, SipC, SopB, SopE, and SopE2, manipulate the host cells cytoskeleton, leading to membrane ruffling and bacterial uptake via a process similar to phagocytosis [151,169,252]. Once inside host cells, *Salmonella* persists within permissive vacuoles by utilizing components encoded on pathogenicity island 2 (SPI-2) [253].

Other studies have implicated caveolin-1 in *Salmonella* internalization [170,171,172]. However, *Salmonella* does not use caveolae for entry host cells but instead induces actin reorganization and membrane ruffling by delivering SopE effector proteins into the host cells via a T3SS [170]. SopE then interacts with the Rho GTPase Rac1 and caveolin-1, leading to membrane ruffling and subsequent bacterial internalization [170].

#### 4.1.7. *Chlamydia*

The genus *Chlamydia* comprises several species, of with *C. trachomatis* is the most common sexually transmitted bacterium, and *C. pneumoniae* a major cause of respiratory infections, also been implicated in atherosclerosis [254]. *Chlamydia* are intracellular pathogens that have evolved effective mechanisms to enter and survive within host cells [255]. Since *Chlamydia* can replicate only inside eukaryotic cells, successful attachment, entry, and evasion of lysosomal degradation are essential stages in their infection cycle [256].

*Chlamydia* infection begins with its attachment to and entry into host cells [257]. The primary targets are the epithelial cells that line the mucosa of the respiratory tract, genital tract, conjunctiva, and gut [258,259,260]. As these cells are non-phagocytic, *Chlamydia* must actively induce its own uptake by modifying the cortical actin cytoskeleton and manipulating the endocytic machinery [261]. This process facilitates the phagocytosis of infectious elementary bodies (EBs) [261]. Uptake begins with the stable attachment of EBs to the epithelial cell surface, involving several bacterial virulence factors and host receptors [255,257,261]. Once adhesion is established, signaling pathways are activated to initiate various host cell processes, including cortical actin reorganization to enable bacterial uptake [257,261]. Several cytoskeleton-related factors are required for *Chlamydia* invasion, including Rac1 and/or Cdc42, phosphatidylinositol 3-kinase (PI3K) and the WAVE regulatory complex [255]. Actin reorganization is associated with extensive membrane remodeling, facilitated by several host factors such as cholesterol-rich lipid rafts, clathrin, and caveolin [173]. Boleti et al. [262] reported a clathrin-independent, dynamin-dependent entry of *Chlamydia* in epithelial cells. *C. trachomatis* has been shown to enter epithelial cells and mouse macrophages via caveolin-containing sphingolipid and cholesterol-enriched raft microdomains [174,175,263]. This pathway is believed to play a crucial role in preventing chlamydial phagosomes from fusing with lysosomes by directing them to the Golgi region [174,263]. Caveolin is then thought to facilitate the interception of exocytic vesicles from the Golgi by chlamydial inclusions [174,263].

#### 4.1.8. Other Bacterial Pathogens

Other pathogens shown to internalize into host cells via a CIE pathway include *Helicobacter pylori* [176], several *Brucella* species [139,177,264], *Campylobacter jejuni* [178], and *Francisella tularensis* [179].

### 4.2. Exploitation of CIE by Viral Pathogens

As obligate intracellular parasites, viruses depend entirely on host cells to complete their life cycle. A critical stage in this process is the initial phase of infection, known as entry, during which viruses deliver their genetic material to the appropriate site for replication. Viruses have evolved various strategies to enter host cells, often bypassing the well-characterized CME pathway. Thus, many viruses use CIE pathways to evade immune detection, enhance infectivity, and target specific intracellular compartments. These alternative pathways include caveolin-mediated endocytosis and lipid raft-mediated uptake [265,266]. By exploiting these pathways, viruses provide valuable insights into the molecular and cellular mechanisms governing these specialized forms of endocytosis [266]. Key examples of viral pathogens that use CIE to invade host cells are highlighted in the following sections (Table 2).

#### 4.2.1. Simian Virus 40 (SV40)

**Figure 7:**
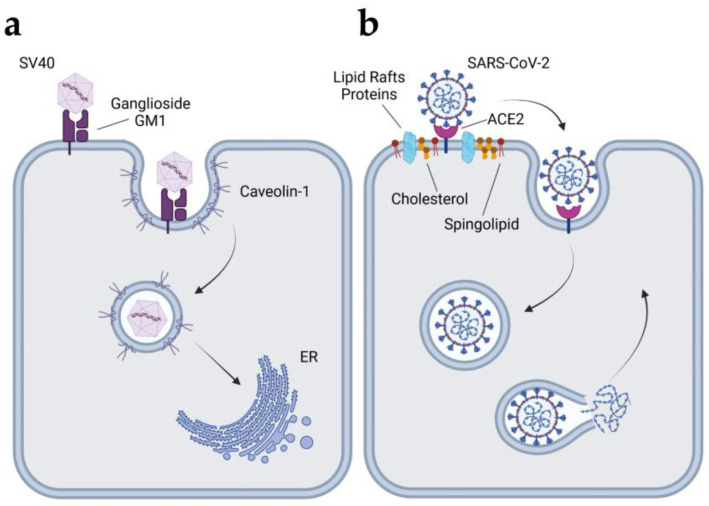
CIE pathways used by SV40 and SARS-CoV-2 to invade host cells. (**a**) SV40 binds to ganglioside GM1 receptors on the host cell surface. The virus is internalized via caveolae-mediated endocytosis, transported to the ER, and subsequently penetrates the cell cytosol before moving to the nucleus. (**b**) SARS-CoV-2 primarily uses ACE2, embedded in lipid rafts, as its main receptor to enter host cells. SV40, simian virus 40; ER, endoplasmic reticulum; SARS-CoV-2, severe acute respiratory syndrome virus 2; ACE2, angiotensin-converting enzyme 2. Created with BioRender.com.

Simian virus 40 (SV40) is a DNA virus that has been extensively used to study the caveolae-mediated entry pathway. The first step in SV40 life cycle involves adhesion to host cells. The virus is thought to recognize its target cells primarily by binding to the ganglioside GM1 via the VP1 protein of the viral capsid [279,280]. The entry of the virion into the cell is mediated by caveolin-mediated endocytosis [62,267] (Figure 7a). The endosome containing SV40 targets the virion to the endoplasmic reticulum (ER), where it undergoes structural changes before penetrating into the cell cytosol [281]. The virion is then transported into the nucleus, where early transcription is initiated [282]. Major histocompatibility class I molecules serve also as specific cell surface receptors for SV40 [283].

#### 4.2.2. Echoviruses

Enteric cytopathic human orphan virus (Echovirus) is a type of small RNA virus that can cause a range of mild diseases in humans, primarily in the intestinal tract. In some cases, however, it can cause serious diseases, such as aseptic meningitis, particularly in young children, people with weakened immune systems, or those with underlying medical conditions [284]. The different steps of echovirus infection involve attachment to the host cell via a surface receptor, followed by internalization into the host cell and uncoating, which releases the RNA from the capsid into the cell cytoplasm. Several echoviruses use CIE to enter host cells [268,285]. For example, it has been shown that after binding to the α2β1 integrin, echovirus 1 is rapidly internalized via caveolae into CV-1 cells [269]. The internalization process is dependent on cholesterol, dynamin-2, and phosphorylation events, and does not require cytoskeletal reorganization [269].

#### 4.2.3. Coronavirus

Coronaviruses are a large class of RNA viruses with an envelope that can cause infections ranging from the common cold to more severe diseases, such as severe acute respiratory syndrome (SARS) [286]. The best-known coronavirus in recent years is SARS-CoV-2, which causes coronavirus disease 2019 (COVID-19) [287]. SARS-CoV enters the host cells via a CIE pathway that involves lipid rafts [270,271]. The virus enters host cells through its transmembrane spike glycoprotein (S protein), which extends from the viral surface [288]. SARS-CoV-2 primarily uses angiotensin-converting enzyme 2 (ACE2) as its main receptor to enter host cells [289,290,291] (Figure 7b). ACE2 is embedded in lipid rafts and plays a crucial role in the initial stage of infection [272].

Human coronavirus 229E (HCoV-229E) is one of the coronaviruses that cause the common cold in humans [292]. CD13 (Aminopeptidase N) has been identified as the receptor for HCoV-229E [293]. Once attached to CD13, HCoV-229E can enter the cell through caveolae-dependent endocytosis, facilitated by caveolin-1 [273].

Human coronavirus OC43 (HCoV-OC43) is a strain of coronavirus that typically causes mild upper respiratory tract infections. However, it has also been shown to have neuroinvasive properties and can lead to severe disease and fatal pneumonia, particularly in children, the elderly, and immunocompromised individuals [294,295]. HCoV-OC43 has been reported to use HLA class I molecule or sialic acids as receptors [296], and caveolin-1-dependent endocytosis to enter the host cell [274]. The scission of virus-containing vesicles from the cell surface has been shown to be dynamin-dependent [274].

#### 4.2.4. Human Immunodeficiency Virus 1 (HIV-1)

The human immunodeficiency virus 1 (HIV-1) is an enveloped retrovirus that primarily buds from the plasma membrane of infected T cells. It has been reported that viral assembly and budding occur at lipid rafts on infected cells [275,276]. In particular, the viral Gag protein has been shown to specifically associate with lipid rafts at the plasma membrane [297].

#### 4.2.5. Japanese Encephalitis Virus (JEV)

Japanese Encephalitis Virus (JEV) is a mosquito-borne virus that causes Japanese encephalitis, a severe infection of the brain [298]. The entry of JEV into B104 rat neuroblastoma cells has been reported to be dynamin-dependent and caveolae-mediated, but independent of clathrin [278]. Binding of JEV to the host cell triggers the EGFR-PI3K signaling pathway, leading to activation of RhoA, which in turn induces phosphorylation of caveolin-1 [277]. Subsequent activation of Rac1 promotes caveolin-associated viral internalization [277].

## 5. Conclusions and Future Research Directions

CIE is used by many different pathogens to enter the host cells. Internalization via CIE generally bypasses the classical endosome-lysosome pathway and avoids intracellular degradation, thereby enabling pathogen survival. For extracellular pathogens, internalization into non-phagocytic host cells via CIE may provide a means to evade elimination by phagocytic cells and avoid the killing effect of antibiotics that have restricted access to the intracellular compartment. Intracellular pathogens can use CIE to gain access to an intracellular niche that is permissive for bacterial survival and replication. By using CIE, viruses can evade immune detection, manipulate host signaling pathways, and create a more favorable environment for replication. Despite the diversity of their structures and life cycles, mechanisms of pathogenesis, and clinical presentations, both bacteria and viruses have evolved to use CIE as a common pathway to enter host cells. This convergence highlights the importance of understanding CIE for the development of broad-spectrum therapeutic strategies that can target multiple pathogens by disrupting their entry mechanisms.

Several open questions remain in this area of research. For instance, many of the virulence factors that pathogens use to manipulate CIE pathways for entering host cells have yet to be identified. Discovering these factors is crucial for developing targeted inhibitors that block pathogen internalization without interfering with essential host cell endocytosis. Furthermore, growing evidence suggests that different stages of the pathogen life cycle may favor distinct entry mechanisms. This raises the question: How do CIE-based entry mechanisms vary across different stages of the pathogen life cycle?

The route a pathogen takes to enter a host cell can have profound consequences, particularly in terms of immune evasion, replication success, and immune system detection. Pathogens that enter host cells via CIE often bypass classical pattern recognition receptor (PRR) checkpoints, especially Toll-like receptors (TLRs) that localize to endosomes. By avoiding traditional endocytic routes, pathogens may delay or reduce antigen processing and presentation, enabling evasion of early immune responses. Therefore, it is also important to investigate how the route of entry influences subsequent immune responses, including antigen presentation.

Answering these questions will require interdisciplinary approaches that combine cell biology, immunology, and computational modeling. Cutting-edge technologies are improving our ability to study how pathogens use CIE to invade host cells. Advanced imaging techniques, including super-resolution microscopy and live-cell tracking, now allow researchers to observe the process of pathogen entry in real time with remarkable clarity. High-throughput screening and CRISPR-mediated gene editing are helping to discover critical host components involved in CIE. Meanwhile, artificial intelligence and computational modelling are offering deeper insights into the complex interactions between pathogens and their hosts. These technological breakthroughs are significantly advancing our understanding of CIE mechanisms and paving the way for innovative treatments for infectious diseases. By targeting the molecular machinery of CIE, it may be possible to develop interventions that block or reverse pathogen entry into host cells. Such approaches could offer powerful tools to combat infections, especially those caused by microbes that have developed resistance to conventional therapies.

## Figures and Tables

**Table 1:** Examples of bacterial pathogens that use CIE for internalization into host cells.

Bacteria	Cell Type	CIE Pathway	References
*Listeria monocytogenes*	Epithelial cells	Caveolin-1-mediated	[153,154]
*Mycobacterium tuberculosis*	Macrophages	Cholesterol-rich domains	[155,156]
	Mast cells	Lipid rafts	[157]
BCG	Macrophages	Caveolin-1-mediated	[158]
*Streptococcus pyogenes*	Epithelial cells (HEP-2)	Caveolae-mediated	[159]
*Staphylococcus aureus*	Human respiratory epithelial cells	caveolin-1- and cholesterol-rich lipid rafts	[160]
*Escherichia coli*	Human bladder epithelial cells	Rho-family GTPases-mediated	[161]
		Focal adhesion and Src family kinases	[162]
		Caveolae/lipid raft	[163,164,165]
	Macrophages	Lipid-rich microdomains	[166]
	Mast cells	Caveolae	[167]
	Human brain microvascular endothelial cells	Caveolae	[168]
*Salmonella typhimurium*	Epithelial cells (HEP-2)	Macropinocytosis	[169]
	Senescent human diploid fibroblasts	Caveolae	[170,171]
	Human M cells	Caveolae	[170,172]
*Chlamydia*	Epithelial cells	Cholesterol- and sphingomyelin-rich plasma membrane microdomain	[173,174,175]
	Macrophages	Cholesterol- and sphingomyelin-rich plasma membrane microdomain	[174]
*Helicobacter pylori*	Gastric epithelial cells	Undefined CIE	[176]
*Brucella*	Macrophages	Lipid rafts	[139,177]
*Campylobacter jejuni*	Intestinal epithelial cells	Caveolae-mediated	[178]
*Francisella tularensis*	Macrophages	Lipid rafts	[179]

**Table 2:** Examples of viral pathogens that use CIE for internalization into host cells.

Virus	Cell Type	CIE Pathway	References
Simian virus 40 (SV40)	African green monkey fibroblast cells (CV-1)	Caveolae-mediated	[62,267]
*Echoviruses*	Primary osteosarcoma cells (Saos cells)	Caveolae-mediated	[268,269]
	African green monkey fibroblast cells (CV-1)	Caveolae-mediated	[269]
Severe acute respiratory syndrome coronavirus 2 (SARS-CoV-2)	293E-ACE2-Myc cells	Cholesterol- and sphingolipid-rich lipid raft microdomains	[270]
	African green monkey fibroblast cells (Vero E6 cells)	Lipid rafts	[271,272]
Human coronavirus 229E (HCoV-229E)	Fibroblasts	Caveolae-mediated	[273]
Human coronavirus OC43	Human ileocecal colorectal adenocarcinoma (HCT-8 cells)	Caveolin-1-mediated	[274]
Human immunodeficiency virus 1 (HIV-1)	MT-2 cells	Membrane raft microdomains	[275]
	Jurkat cells	Lipid rafts	[276]
Japanese Encephalitis Virus (JEV)	Human neuronal cells	Caveolin-1-mediated	[277]
	B104 rat neuroblastoma cells	Caveolae-mediated	[278]

## Data Availability

No new data were created or analyzed in this study.

## Abbreviations

The following abbreviations are used in this manuscript:

CME	Clathrin-mediated endocytosis
CIE	Clathrin-independent endocytosis
EGF	Epidermal growth factor
EGFR	Epidermal growth factor receptor
Arf6	ADP-ribosylation factor 6
ER	Endoplasmic reticulum
RhoA	Ras homolog family member A
Rac1	Rac Family Small GTPase 1
Cdc42	Cell division control protein 42
RhoG	Ras homolog family member G
IL-2R-β	Interleukin-2 receptor subunit beta
GPI-Aps	Glycosylphosphatidylinositol-anchored proteins
FEME	Fast endophilin-mediated endocytosis
SH3	Src homology 3
BAR	Bin-Amphiphysin-Rvs domain
CIP4	Cdc42-interacting protein 4
FBP17	formin-binding protein 17 GTPase-activating proteins
SHP2	SH2-containing inositol phosphatase 2
Cdk5	Cyclin-dependent kinase 5
GSK3β	Glycogen synthase kinase-3 beta
RTN3	Reticulon-3
IP3R	Inositol triphosphate receptor
IRSp53	Insulin receptor substrate p53
CLIC/GEEC	Clathrin-independent carrier (CLIC)/GPI-anchored protein-enriched early endocytic compartments (GEEC)
GPI-Aps	GPI-anchored proteins
PI3K	Phosphoinositide 3-kinases
GTP	Guanosine triphosphate
GDP	Guanosine diphosphate
GBF1	Golgi Brefeldin A Resistant Guanine Nucleotide Exchange Factor 1
GEF	Guanine nucleotide exchange factors
Arf1	ADP-ribosylation factor 1
ARHGAP10	Rho GTPase Activating Protein 10
GRAF1	GTPase Regulator Associated with Focal Adhesion Kinase 1
GAPs	GTPase-activating proteins
PIP_2_	phosphatidylinositol 4,5-bisphosphate
FME	Flotillin-mediated endocytosis
SPFH	stomatin/prohibitin/flotillin/HflK/C
InlA	Internalin A
InlB	Internalin B
PlcA	Phosphatidylinositol-Specific Phospholipase C
PlcB	Broad-Range Phospholipase C
LIPI-1	Listeria pathogenicity island 1
EHD2	EH Domain Containing 2
LAP	Listeria adhesion protein
Hsp60	Heat shock protein 60
CR3	Complement receptor 3
TACO	Tryptophan-aspartate-containing coat
BCG	*Mycobacterium bovis* Bacillus Calmette-Guérin
SfbI	Streptococcal fibronectin-binding protein I
OCRL	Oculocerebrorenal Syndrome of Lowe
EPEC	Enteropathogenic *E. coli*
EHEC	Enterohaemorrhagic *E. coli*
UPEC	Uropathogenic *E. coli*
UTIs	Urinary tract infections
CNF1	Cytotoxic necrotizing factor 1
UP1a	Glycosylated uroplakin Ia
CEACAM	Carcinoembryonic antigen-related cell adhesion molecule
IBCs	Intracellular bacterial communities
DAEC	Afa/Dr diffusely adhering *E. coli*
PKCa	Protein kinase C alpha
T3SS	Type III secretion system
SPI-1	Salmonella Pathogenicity Island 1
SCVs	*Salmonella*-containing vacuoles
SPI-2	Salmonella Pathogenicity Island 2
Ebs	Elementary bodies
SV40	Simian virus 40
SARS	Severe acute respiratory syndrome
ACE2	Angiotensin-converting enzyme 2
HIV-1	Human immunodeficiency virus 1
JEV	Japanese Encephalitis Virus
CRISPR	Clustered regularly interspaced short palindromic repeats
